# Incidence and impact of totally occluded culprit coronary artery in patients with non-ST segment elevation myocardial infarction acute coronary syndrome

**DOI:** 10.1186/s43044-021-00160-x

**Published:** 2021-04-07

**Authors:** Sherif W. Ayad, Tarek H. El Zawawy, Mohamed I. Lotfy, Ahmed M. Naguib, Ahmed M. El Amrawy

**Affiliations:** Department of Cardiology, Faculty of Medicine, Alexandria University, Alexandria, Egypt

**Keywords:** Non-ST segment elevation myocardial infarction, Coronary total occlusion, Electrocardiography

## Abstract

**Background:**

Significance of totally occluded culprit coronary artery in patients presenting with non-ST segment elevation myocardial infarction (NSTEMI) is underestimated. The aim of the study was to evaluate the incidence and impact of totally occluded culprit artery on in-hospital and 6 months follow-up outcomes of NSTEMI acute coronary syndrome (ACS) patients.

**Results:**

We collected retrospectively data of 500 NSTEMI patients who presented to our hospital from June 2016 to June 2017. All patients underwent PCI within 72 h of presentation. We excluded patients with cardiogenic shock, prior CABG, and STEMI. Patients were divided into two groups according to pre-procedural culprit vessel thrombolysis in myocardial infarction (TIMI) flow. Group 1, TIMI flow 0 total coronary occlusion, included 112 patients (22.4%). Group 2, TIMI flow 1–3 non-total occlusion, included 388 patients (77.6%). Group 1 patients had significantly higher incidence of smoking (*p*=0.01), significantly higher level of cardiac enzymes (*p*<0.001), significantly more collaterals (*p*<0.001), and significantly more LCX and RCA as the culprit vessel (*p*<0.01), while group 2 patients had significantly higher incidence of diabetes (*p*=0.02) and significantly more LAD as the culprit vessel. There were no significant differences between the two groups regarding the major adverse cardiac and cerebrovascular events (MACCE) in-hospital (5.3% in totally occluded group vs. 1% in non-totally occluded group, *p*=0.07), but group 1 patients had significantly higher incidence of in-hospital arrhythmia (8.9% in group 1 vs. 1% in group 2, *p*=0.007). After 6 months follow-up, there were no significant differences regarding MACCE between the 2 groups after 6 months follow-up (5.4% in group 1 vs. 4.6% of group 2, *P*=0.24).

**Conclusion:**

22.4% of NSTEMI patients have a totally occluded culprit artery. The presence of an occluded culprit artery did not significantly affect the clinical outcomes of NSTEMI patients either in-hospital or after 6 months follow-up but was associated with significantly higher incidence of in-hospital arrhythmia.

## Background

The spectrum of acute coronary syndrome (ACS) including ST-segment elevation myocardial infarction (STEMI), non-ST segment elevation myocardial infarction (NSTEMI), and unstable angina have become the leading cause of death globally [1–3].

In the Middle East including Egypt, 64% of ACS patients present with NSTEMI and 36% present with STEMI according to the results of the ACCESS registry [4].

Recent studies have shown that the incidence of NSTEMI has slightly increased over the last decade and have lower short-term mortality compared with STEMI patients, while at 1 or 2 years follow-up mortality rates become higher [5].

Based on electrocardiogram (ECG), ACS patients can be divided into STEMI patients with ST-segment elevation on ECG denoting total or near total occlusion of the culprit artery and necessitating immediate coronary angiography and revascularization or NSTEMI patients with ST-segment depression or T-wave inversion on ECG with early angiography and revascularization only if they have high risk [6, 7].

Previous studies have shown that nearly 25% of NSTEMI patients present with a totally occluded coronary artery, and two-thirds of the occlusions are already collateralized at the time of angiographic examination [8, 9]; this was more common in patients presenting with either right coronary artery (RCA) or left circumflex artery (LCX) involvement [8–14], which could be explained by the lack of ECG sensitivity in detecting acute ischemia in the inferolateral and posterior walls.

The lack of classic ST-segment elevation on ECG in these subset of NSTEMI patients, despite the presence of totally occluded culprit artery, lead to either delay in or no revascularization [15].

Currently, the impact of totally occluded artery on the outcome of NSTEMI patients is still unclear, as previous studies had reported contradictory results. Some studies reported worse outcome for patients with totally occluded culprit artery [9, 13, 16] while other studies reported no significant difference in outcome [8, 10, 11, 17].

In this study, we aimed to evaluate the incidence and impact of totally occluded culprit artery on in-hospital and after 6 months follow-up outcomes of NSTEMI patients.

## Methods

### Study design

This is a retrospective observational study conducted on consecutive NSTEMI patients who presented to our hospital between 1 June 2016 and 1 June 2017.

The inclusion criteria were established diagnosis of NSTEMI (patients with acute chest pain but no persistent ST-segment elevation, ECG changes may include transient ST-segment elevation, persistent or transient ST-segment depression, T-wave inversion, flat T waves, pseudo-normalization of T waves or the ECG may be normal with elevated cardiac biomarkers) and fulfilling guideline recommendation for PCI treatment [7] with invasive strategy performed within 72 h of admission. The exclusion criteria were previous CABG, cardiogenic shock, previous PCI of same culprit vessel, STEMI, and left main coronary artery disease. The study population included 500 patients. This is an all-comer study, and therefore, a formal sample size calculation was not required.

### Data collection

All patients’ demographic data were collected including age, gender, comorbidities (hypertension, diabetes, dyslipidemia, prior ACS or PCI), and smoking.

From laboratory data, we registered troponin I, creatine kinase MB fraction (CK-MB), urea, and creatinine levels on admission and peak levels during the hospital stay.

ECG and echocardiographic data including ejection fraction (EF), wall motion abnormalities (WMA), and degree of mitral regurgitation (MR) were also registered.

Among in-hospital treatments, we registered PCI procedure details including access site, procedure outcomes, complications, and the use of antithrombotic therapy (acetyl salicylic acid, clopidogrel, ticagrelor, heparin, enoxaparin, and glycoprotein IIb/IIIa inhibitors).

The culprit artery was identified primarily on angiographic findings with ECG and echocardiogram to support the assessment if needed. Totally occluded artery was defined as a lesion with 100% stenosis and a thrombolysis in myocardial infarction (TIMI) flow 0 [18].

Baseline and at hospital discharge GRACE and TIMI risk scores were calculated [19, 20].

Baseline syntax score and residual syntax score were also calculated [21].

### Clinical endpoint measurements

The primary clinical outcomes of the study were MACCE which was defined as a composite of death, re-infarction, need for revascularization, heart failure, and cerebrovascular stroke either in-hospital or after 6 months follow-up.

### Statistical analysis

Data were analyzed using the Statistical Package for Social Sciences (SPSS ver.20 Chicago, IL, USA) [22]. K-S test of normality was done to check for normality of quantitative variables. Normally distributed data were described using mean and standard deviation, while not normally distributed data were described using median and range, and non-parametric tests were applied. We used number and percent to describe qualitative data. Independent Student *t*-test was used to compare age between the 2 groups. Mann-Whitney *U* test was used to compare non-parametric quantitative parameters between the 2 groups. Pearson Chi square was used to compare 2 × 2 categorical variables, and Fisher’s exact test if >20% of cells had expected cell count less than 5. And in > 2×2 table, we used Monte Carlo significance test if >20% of cells had expected cell count less than 5. Kaplan-Meier survival curve was done for survival analysis of MACCE occurrence, with duration in days till occurrence; Log rank test used was to compare survival between the 2 groups. Any test was considered significant below or equal to 0.05.

An informed consent was obtained from every patient or the legal guardians. The study was approved by the local ethics committee.

## Results

### Patient characteristics

The study population included 500 patients who were classified into two groups:

- Group 1 included 112 patients with totally occluded culprit vessel.
- Group 2 included 388 patients with non-totally occluded culprit vessel.

Both patient groups (1 and 2) were well matched with respect to demographic data and clinical characteristics with no significant difference between them except for smoking which was significantly higher in group 1 (55.3% vs. 44.3%, *p*=0.01) and diabetes mellitus (DM) which was significantly higher in group 2 (25% vs. 41.7%, *p*=0.02). Regarding the mean time from admission to PCI, there was no statistically significant difference between the two groups (27.11 ± 10.6 h vs. 28.7 ± 11.9 h, *p*=0.35). The baseline characteristics of both groups are presented in Table 1.

**Table 1:** Baseline characteristics of the studied populations

	**Group 1 (*n*=112)**		**Group 2 (*n*=388)**		**Total sample**	**Test value (*p*)**
**Age**						
**Mean ± SD (years)**	56.54 ± 9.437		59.09 ± 9.686		57.53 ± 9.57	*p*=0.08
**Sex**						
Male	98	87.5%	302	77.8%	400 (80%)	*p*=0.11
Female	14	12.5%	86	22.2%	100 (20%)	
**DM**						
**Insulin treated**	8	7.14%	30	7.74%	228 (45.6%)	^*FE^***p*=0.02**
**Orally treated**	28	25%	162	41.7%		
**HTN**	56	50%	244	62.9%	300 (60%)	*p*=0.08
**Smoker**						
**Current smoker**	62	55.3%	172	44.3%	234 (46.8%)	^* FE^***p*** **=0.01**
**Former smoker**	18	16.1%	30	7%	48 (9.6%)	
**Dyslipidemia**	44	39.3%	120	30.9%	164 (32.8%)	*p*=0.24
**CKD**	4	3.6%	36	9.3%	40 (8%)	*p*=0.262
**FH of CAD**	24	21%	70	18%	94 (18.8%)	*p*=0.56
**Hx of ACS**	4	3.6%	32	8.2%	36 (7.2%)	*p*=0.378
**Hx of PCI**	8	7.1%	56	14.4%	64 (12.8%)	*p*=0.15
	**Group 1 (*n*=112)**			**Group 2 (*n*=388)**		***p***
**Admission to PCI**						
Min.—Max. **(h)**	12–72			12–72		^MW^*p* = 0.35
Mean ± SD. **(h)**	27.11 ± 10.651			28.73 ± 11.956		
Median **(h)**	24			24		
**Killip class**						
**I**	102		91.07%	362	93.3%	^MW^*p*=0.77
**II**	6		5.4%	18	4.6%	
**III**	4		3.6%	8	2.1%	

*χ*^2^ value for Chi square, *t* Student *t*-test, *MW* Mann-Whitney test, *FEP* Fisher’s exact significance

*Statistically significant at *p* ≤ 0.05

### ECG, echocardiography, and laboratory results

CK-MB and troponin I levels were significantly higher in group 1 than in group 2 (92 vs. 35ng/ml, *p*<0.001 and 7.5 vs. 1.5ng/ml, *p*<0.001). There was no significant difference between the two groups regarding other laboratory, ECG, and echocardiography results. The baseline ECG, echocardiography, and laboratory results of both groups are presented in Table 2.

**Table 2:** Baseline ECG, echocardiography, and laboratory results of both groups

	Group 1 (***n***=112)		Group 2 (***n***=388)		***p***
	No.	%	No.	%	
**Initial ECG**					
Normal	64	57.1	238	61.3	*p* = 0.434
ST depression	30	26.8	66	17.0	*p* = 0.102
T-wave inversion	8	12.5	56	14.4	*p* = 0.7
Q waves	2	1.8	22	5.7	*p* = 0.3
Bundle branch	2	1.8	6	1.5	*p* = 1
**Hb**					
Min.–Max. **(g/dl)**	9.0–15.0		11.0–16.0		^t^*p* = 0.697
Mean ± SD. **(g/dl)**	13.20 ± 1.66		13.36 ± 1.44		
Median **(g/dl)**	13.0		13.25		
**S.creatinine**					
Min.–Max. **(mg/dl)**	0.60–3.20		0.60–1.70		^MW^*p*=0.857
Mean ± SD. **(mg/dl)**	1.03 ± 0.50		0.95 ± 0.23		
Median **(mg/dl)**	0.90		0.95		
**CKMB**					
**Median (ng/ml)**	92.0000		35.0000		*p=<0.001
**(Min–max) (ng/ml)**	(5.0–125)		(5.5–116)		
**Troponin**					
**Median(ng/ml)**	7.5000		1.5000		*p=<0.001
**(Min-Max) (ng/ml)**	(0.5–35)		(0.5–45)		
**ECHO**					
**EF**					
Min-Max (%)	35–75		30–70		*p*=0.344
Mean ± SD (%)	58.64 ± 7.44		59.19 ± 7.616		
Median (%)	60		60		
**RWMAS**	52	46.4%	140	36.1%	*p* =0.16
**Mitral regurgitation**					
No	92	82.1%	310	79.9%	^MW^*p*=0.492
Mild	20	17.9%	62	16%	
Moderate	0	0%	12	3.1%	
Severe	0	0%	4	1.5%	

*χ*^2^ value for Chi square, *t* Student *t*-test, *MW* Mann-Whitney test

*Statistically significant at *p* ≤ 0.05

### GRACE risk score and TIMI risk score

There were no statistically significant differences between the two groups as regard the GRACE risk score at admission or TIMI risk score at admission (Table 3).

**Table 3:** GRACE risk score and TIMI risk score at admission of the studied population

	Group 1 (***n*** = 112)		Group2 (***n*** = 388)		Test of significance	***p***
	No.	%	No.	%		
**GRACE risk score** [19]						
Low (1–108)	64	57.3	232	59.8	***χ***^**2**^ **=**2.67	^**MC**^***p*** =0.28
Intermediate (109–140)	40	35.7	114	31.9		
High (141–372)	8	7	32	8.3		
**TIMI risk score** [20]						
Low (0–2)	20	17.8	80	20.6	***χ***^**2**^**=**1.567	^**MC**^***p*** =0.37
Intermediate (3–5)	82	73.3	268	69.1		
High (>5)	10	8.9	40	10.3		

*χ*^2^ value for Chi square, *MC* Monte Carlo test

*Statistically significant at *p* ≤ 0.05

### Procedural characteristics of the studied population

With regard to the angiographic data, the incidence of multivessel disease was not different between the two groups; group 1 patients had significantly higher percentages of LCX and RCA as the culprit vessel (55.4% and 23.2%), while group 2 patients had significantly higher percentages of LAD as the culprit vessel (53.1%). This was statistically significant *p*<0.001. Also, there was no significant difference between the two groups regarding the baseline syntax score (11.68 ± 6.05 vs. 6.79 ± 3.24, *p* =0.12) or the residual syntax score (1.27 ± 2.67 vs. 0.66 ± 2.35, *p* =0.07). The presence of collaterals was significantly higher in group 1 than in group 2 (73.2% vs. 4.6%, *p*<0.01).

All patients in the two groups were done through transfemoral approach and received drug-eluting stents (DES), and no patient had procedure-related complications. Also, the antiplatelet treatment with clopidogrel or ticagrelor did not differ, but the use of GP IIb/IIIa inhibitors was significantly higher in group 1 (23% vs. 3%, *p*=0.04). All data of the procedural characteristics of the studied population are summarized in Table 4.

**Table 4:** Procedural characteristics of the studied population

	Group 1 (***n***=112)		Group 2 (***n***=388)		*p* value
Number of diseased vessels					
One vessel CAD	32	28.6%	160	41.2%	X2p=0.218
Two vessel CAD	52	46.4%	154	39.7%	
Three vessel CAD	28	25%	74	19.1%	
Culprit vessel					
LAD	24	21.4%	206	53.1%	X2p=<0.001
LCX	62	55.4%	154	25.8%	
RCA	26	23.2%	74	21.1%	
Site of the lesion					
Proximal	28	50%	95	48.9%	X2p=0.23
Mid	20	35.7%	74	37.2%	
Distal	8	14.3%	25	13.9%	
TIMI pre procedure					
0	112	100%	0	0 %	X2p =<0.001
I	0	0%	0	0 %	
II	0	0%	64	16.5%	
III	0	0%	324	83.5%	
Use of GP IIa–IIIb antagonists	26	23%	14	3	*p*=0.04
Collaterals	82	73.2%	18	4.6%	X2p =<0.01
TIMI III post procedure	110	98.2%	384	98.9%	*p*=0.36
Syntax score					
Mean ± SD	11.68 ± 6.05		6.79 ± 3.24		MWp =0.12
Median	10		7.0		
Min–Max	4.0–24.0		4.0–22.0		
Residual syntax score					
Mean ± SD	1.27 ± 2.67		0.66 ± 2.35		MWp=0.07
Median	0		0		
Min–Max	0–15		0–20		

*χ*^2^ value for Chi square, *MW* Mann-Whitney test

*Statistically significant at *p* ≤ 0.05

### In-hospital outcomes

Group 1 patients showed higher risk of cumulative MACCE than group 2 (5.3% versus 1%), but this was not statistically significant (*p*=0.07). Two patients in each group died while in-hospital mostly because of arrhythmia (ventricular fibrillation). The need for revascularization and the incidence of heart failure, re-infarction, ischemic cerebrovascular stroke (CVS), or bleeding were not different between groups. In-hospital arrhythmia was significantly higher in group 1 compared to group 2 (8.9% vs. 1%, *p*=0.007). The data of in-hospital outcomes are summarized in Table 5.

**Table 5:** In-hospital outcomes of the studied population

	Group 1 (***n***=112)		Group 2 (***n***=388)		***p***
	No.	%	No.	%	
**Arrhythmias**	10	8.9	4	1	^*FE^*p*=0.007
AF	4	3.5	2	0.5	
VF	6	5.4	2	0.5	
**HF**	4	3.5	16	0.04	^FE^*p* =1
**Bleeding**	6	5.4	2	0.5	^FE^*p* =0.06
Major	2	1.8	0	0.0	
Minor	4	3.6	2	0.5	
**CIN**	8	7.1	20	5.1	^FE^*p* =0.34
**Local vascular complication**	10	8.9	16	4.1	^FE^*p* =0.22
**MACCE**	**6**	**5.3**	4	**1.0**	^FE^*p* =0.07
**Death**	2	1.8	2	0.01	^FE^*p* =0.**39**
**Reinfarction**	0	0.0	0	0	
**Repeated revascularization**	2	1.8	0	0	^FE^*p* =0.22
**Stroke**	2	1.8	1	0.01	^FE^*p* =0.**39**

^*FE*^*p* Fisher’s exact significance

*Statistically significant at *p* ≤ 0.05

### Six months follow-up

**Fig. 1:**
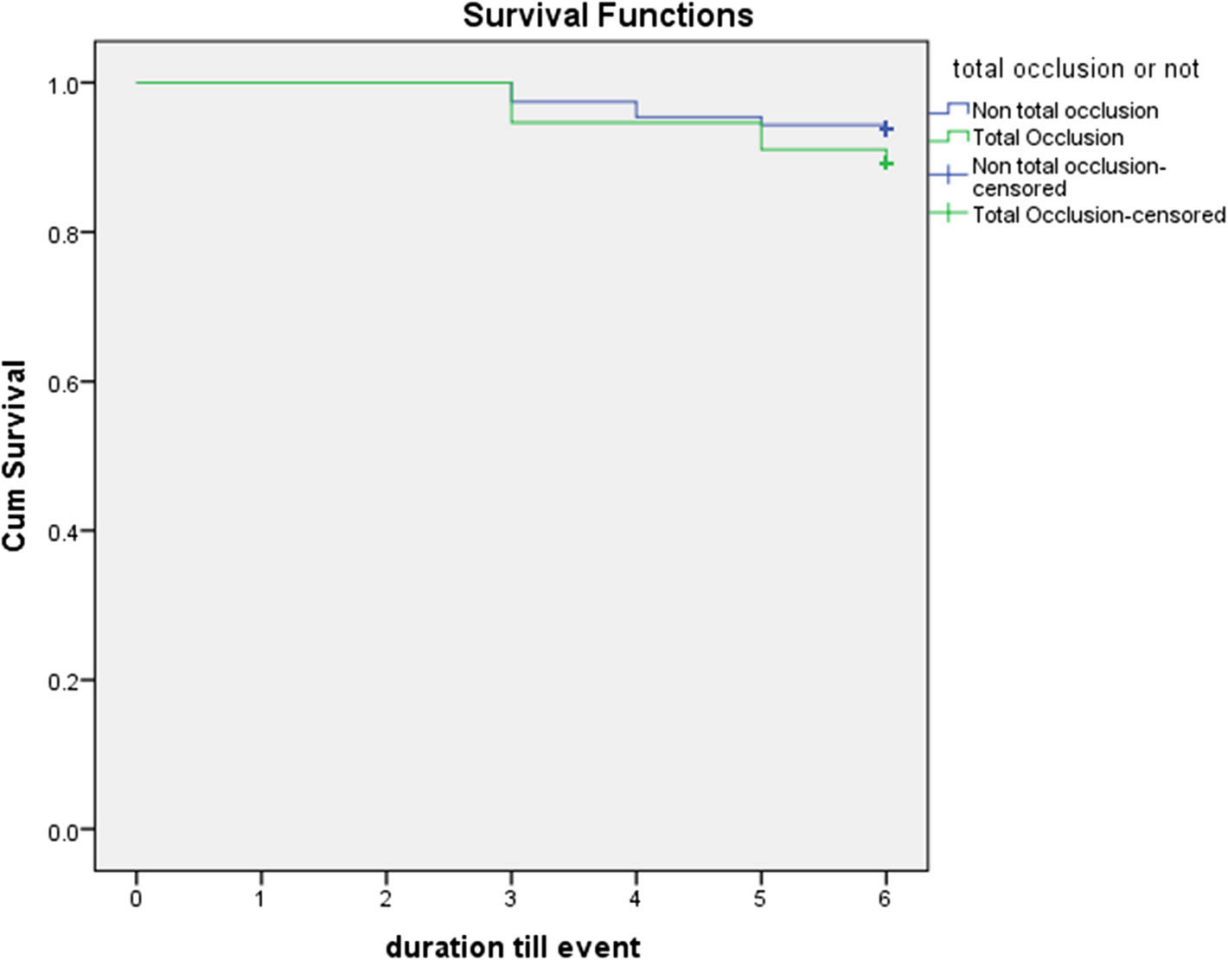
Kaplan-Meier estimates of MACCE between the two groups at 6 months

The mean follow-up in our study was 215± 29.5 days. There was no significant difference between the two groups regarding the composite MACCE endpoint after 6 months follow-up (5.4% vs. 4.6%, *p*=0.24). Two patients in group 1 and 6 patients in group 2 died during follow-up (1.8% vs. 0.02%, *p*=1). The need for revascularization and the incidence of heart failure, re-infarction, ischemic cerebrovascular stroke (CVS), or bleeding were not different between groups. The data of 6 months follow-up outcomes are summarized in Table 6 and Fig. 1.

**Table 6:** Six months follow-up outcomes of the studied population

	Group 1 (***n***=112)		Group 2 (***n***=388)		***p***
	No.	%	No.	%	
**MACCE**	6	5.4	18	4.6	*p*=0.24
**Death**	2	1.8	6	0.02	^FE^*p*=1
**Reinfarction**	2	1.8	8	0.02	^FE^*p*=1
**Repeated revascularization**	2	1.8	4	0.01	^FE^*p*=0.53
**Major bleeding**	0	0	0	0	
**Heart failure**	2	1.8	6	0.02	^FE^*p*=1
**Stroke**	0	0	0	0	

^*FE*^*p* Fisher’s exact significance

*Statistically significant at *p* ≤ 0.05

## Discussion

The presence of an occluded culprit artery in NSTEMI patients cannot be diagnosed based on the clinical or electrocardiographic findings. The rationale beyond the absence of characteristic ST-segment elevation despite totally occluded artery in NSTEMI patients is still not understood. The lack of sensitivity of standard 12-lead ECG to detect changes of total occlusion in the inferolateral distribution [23–25], the presence of good collaterals, acute total occlusion in a territory with dual blood supply, and chronic total occlusion misclassified as acute occlusion could be possible mechanisms [26].

The objective of this study was to evaluate the incidence and impact of totally occluded culprit artery on in-hospital and midterm follow-up outcomes of NSTEMI patients.

In our study, the incidence of totally occluded culprit in NSTEMI patients was 23% which was similar to previous studies that reported an incidence of 25% [9–11].

The mean time from admission to PCI in the totally occluded culprit artery group in our study was 27.11 ± 10.6 h; this was similar to Kim et al. [13] but contradicted by Soon et al. [14] who reported longer time and Karwowski et al. [12] who reported shorter mean time of chest pain to angiography in totally occluded group.

In this study, the median of CKMB and troponin I levels was significantly higher in the totally occluded artery group (*p*<0.001). This was similar to data from Karwowski et al. [12], Bahrmann et al. [11], and Wang et al. [10] that showed significantly higher CKMB and troponin levels in the totally occluded culprit artery populations.

There was no statistically significant difference in the mean left ventricular ejection fraction percentage (LVEF) between the two groups in our study (58.64 ± 7.44% vs. 59.19 ± 7.61%; *p*= 0.34), while Soon et al. [14] reported significantly lower EF in the totally occluded artery group.

Our study showed that in the totally occluded group, LCX represented the culprit vessel in 55.4% of patients, RCA was the culprit in 23.2%, and LAD was the culprit in 21.4% of the patients. In non-totally occluded group, LAD represented the culprit vessel in 53.1% of patients, LCX was the culprit in 25.8%, and RCA was the culprit in 21.1% of patients. This finding is compatible with other trials. Karwowski et al. [12] reported that in the totally occluded group, LCX represented the culprit vessel in 48.1% of patients and RCA in 29.5% of the patients.

The incidence of MACCE either in-hospital or after six months follow-up was similar among both groups, but the incidence of in-hospital arrhythmia was significantly higher in the totally occluded group (*p*=0.007). We do not have a clear explanation for that but we can hypothesize that patients were on full medical therapy (dual antiplatelets, high-intensity statins, beta blockers, and RAS blockers), and the difference in the residual syntax score was not significant.

These results are consistent with most studies addressing impact of totally occluded culprit artery in NSTEMI patients [8–14].

The current study was limited in several ways. Firstly, the current study was a retrospective, single-center study. Secondly, patients with contraindication to PCI were excluded from the study and finally the relatively small sample size.

## Conclusion

22.4% of NSTEMI patients have a totally occluded culprit artery. The presence of an occluded culprit artery did not significantly affect the clinical outcomes of NSTEMI patients either in-hospital or after 6 months follow-up but was associated with significantly higher incidence of in-hospital arrhythmia. Further studies with bigger sample size and longer follow-up duration are recommended.

## Data Availability

All data analyzed during this research are included in this published article.

## Abbreviations

ACS: Acute coronary syndrome
CABG: Coronary artery bypass grafting
CK-MB: Creatine kinase-MB isoenzyme
ECG: Electrocardiography
GRACE: Global Registry of Acute Coronary Events
LCx: Left circumflex artery
LVEF: Left ventricular ejection fraction
MI: Myocardial infarction
NSTEMI: Non-ST segment elevation acute coronary syndrome
STEMI: ST segment elevation myocardial infarction
PCI: Percutaneous coronary intervention
TIMI: Thrombolysis in myocardial infarction
MACCE: Major adverse cardiac and cerebrovascular events
CVS: Cerebrovascular stroke
RCA: Right coronary artery
LAD: Left anterior descending artery
CIN: Contrast-induced nephropathy
AF: Atrial fibrillation
VF: Ventricular fibrillation
CAD: Coronary artery disease
LM: Left main
Hb: Hemoglobin
DM: Diabetes mellitus
HTN: Hypertension
CKD: Chronic kidney disease
WMA: Wall motion abnormality
